# Health-related quality of life among five-year-old extremely preterm children with motor disorders

**DOI:** 10.1093/eurpub/ckac130.249

**Published:** 2022-10-25

**Authors:** AM Aubert, R Costa, S Johnson, U Ådén, V Pierrat, M Cuttini, AF van Heijst, RF Maier, M Sentenac, J Zeitlin

**Affiliations:** 1 Inserm, INRAE, CRESS, EPOPé Research team, Université Paris Cité, Paris, France; 2 EPIUnit, Instituto de Saude Publica, Universidade do Porto, Porto, Portugal; 3 Department of Health Sciences, University of Leicester, Leicester, UK; 4 Department of Women's and Children's Health, Karolinska Institutet, Stockholm, Sweden; 5 Clinical Care and Management Innovation Research Area, Bambino Gesu Children's Hospital, IRCCS, Rome, Italy; 6 Department of Neonatology, Radboud University Medical Center, Nijmegen, Netherlands; 7 Children's Hospital, University Hospital, Philipps University Marburg, Marburg, Germany

## Abstract

**Background:**

Motor disorders resulting from extremely preterm birth (EPT; <28 weeks’ gestation) can limit daily activities, schooling and social relationships. Cerebral palsy (CP) affects about 10% of children and non-CP movement difficulties (MD) are highly prevalent, although they tend to be under-diagnosed, especially in children without other developmental difficulties. We investigated the association between motor disorders and health-related quality of life (HRQoL) among five-year-old children born EPT.

**Methods:**

We included children at age five from a population-based EPT birth cohort born in 2011-2012 in 11 European countries (N = 1,021). Children without CP were classified using the Movement Assessment Battery for Children - 2nd edition as having significant MD (≤5th percentile of standardised norms) or being at risk of MD (6th-15th percentile). Parents reported on CP diagnoses and HRQoL using the Pediatric Quality of Life InventoryTM. We used linear regression to compare HRQoL scores by motor status adjusting for social characteristics.

**Results:**

Children born EPT with CP, significant MD and at risk of MD had lower adjusted HRQoL total scores [95% confidence intervals] than those without MD: -26.1 [-31.0; -21.2], -9.1 [-12.0; -6.1] and -5.0 [-7.7; -2.3]. Decreases were greater for physical scores: -35.3 [-42.7; -27.9], -11.9 [-16.1; -7.8] and -5.4 [-9.1; -1.6] than psychosocial scores: -20.6 [-25.2; -16.0], -7.4 [-10.3; -4.5] and -4.9 [-7.6; -2.1]. These differences persisted after exclusion of children with other developmental difficulties.

**Conclusions:**

Motor disorders among 5-year-old children born EPT were associated with lower HRQoL, even among children with less severe motor difficulties and without other developmental difficulties.

**Key messages:**

- Among five-year-old children born extremely preterm, severity level of motor disorders was associated with reductions of their health-related quality of life.
- Differences persisted after exclusion of children with other developmental difficulties and among children not receiving motor-related health care services had lower health-related quality of life.

